# Influence of Salt Intake on Association of Blood Uric Acid with Hypertension and Related Cardiovascular Risk

**DOI:** 10.1371/journal.pone.0150451

**Published:** 2016-04-04

**Authors:** Lei Hou, Mingtao Zhang, Wei Han, Yong Tang, Fang Xue, Shaohua Liang, Biao Zhang, Weizhi Wang, Kuliqian Asaiti, Yanhong Wang, Haiyu Pang, Zixing Wang, Yuyan Wang, Changchun Qiu, Jingmei Jiang

**Affiliations:** 1 Department of Epidemiology and Biostatistics, Institute of Basic Medical Sciences Chinese Academy of Medical Sciences / School of Basic Medicine Peking Union Medical College, Beijing, China; 2 The People’s Hospital of Altay Prefecture, Xinjiang, China; 3 Hong Dun Town hospital, Altay Prefecture, Xinjiang, China; IPK, GERMANY

## Abstract

**Background:**

A relationship of blood uric acid (UA) with hypertension and cardiovascular risk is under debate thus salt intake is hypothesized to contribute to such associations.

**Methods:**

In this cross-sectional study, stratified cluster random sampling elicited a sample of 1805 Kazakhs with 92.4% compliance. Hypertension and moderate-or-high total cardiovascular risk (mTCR) were defined according to guidelines. Sodium intake was assessed by urinary sodium excretion. Prevalence ratios (PRs) were used to express associations of UA with hypertension and mTCR.

**Results:**

In the highest tertile of sodium intake in women, the adjusted PRs (95% confidence intervals) of low to high quartiles compared with the lowest quartile of UA, were 1.22(0.78–1.91), 1.18(0.75–1.85), and 1.65(1.09–2.51) for hypertension and 1.19(0.74–1.90), 1.39(0.91–2.11), and 1.65(1.10–2.47) for mTCR (*P* for trend <0.05). However, these findings were not shown for other sodium intake levels. There were similar results in men. PRs markedly increased with a concomitant increase in UA and sodium intake and there was a significant interaction (*P* = 0.010) for mTCR with PRs of 1.69(1.10–2.60) for men and 3.70(2.09–6.52) for women in those with the highest compared with the lowest quartile of UA and tertile of sodium intake. Similar findings were shown for hypertension.

**Conclusions:**

This study implied that a high salt intake may enhance the associations of UA with hypertension and cardiovascular risk.

## Introduction

A relationship between uric acid (UA) and hypertension, which leads to at least 1.5 million cardiovascular (CV) deaths per year [1], has been under debate for decades. Most observational studies have reported that elevated UA levels is likely linked with hypertension in Caucasian [2,3], African-Americans [3], and Chinese Han [4], and particularly with the incident hypertension in adolescents [2,5]. However, some studies have reported no such association in men or the elderly [6,7], in particular the Framingham Study, which thought the UA level was an independent predictor of incident hypertension and longitudinal BP progression [2], but did not indicate a causal role of UA in CV risk [8]. Therefore, the associations of UA with hypertension and related CV risk remain less well established, and UA is not considered as a risk factor for hypertension and CV diseases in current guidelines [1,9,10].

A high-salt intake is well established as the most important risk factor of hypertension. A rat model that hyperuricemia induced salt-sensitivity, which is linked to hypertension and CV risk, has been established [11]. Recently, an increased salt intake was associated with increased UA levels, which enhanced the association of a high-salt diet with hypertension [12]. However, whether a high-salt diet plays a role on the association between UA and hypertension has not been explored.

We hypothesized a high-salt diet contributes to associations of UA with hypertension and related CV risk. The present study explored influence of diet salt on such association in Kazakhs, a people across Eurasia, who are well known to have a high level of sodium intake and prevalence of hypertension.

## Methods

### Study population

Hong Dun town is the base of this study locating in the urban-rural fringe of Altay county-level city in the North Xinjiang, China. 12 Kazakh-based administrative villages and, one township office which almost covers all citizens of this town, were included in this study after excluding villages with less than 100 Kazakh individuals who live together with other ethnic groups.

**Fig 1:**
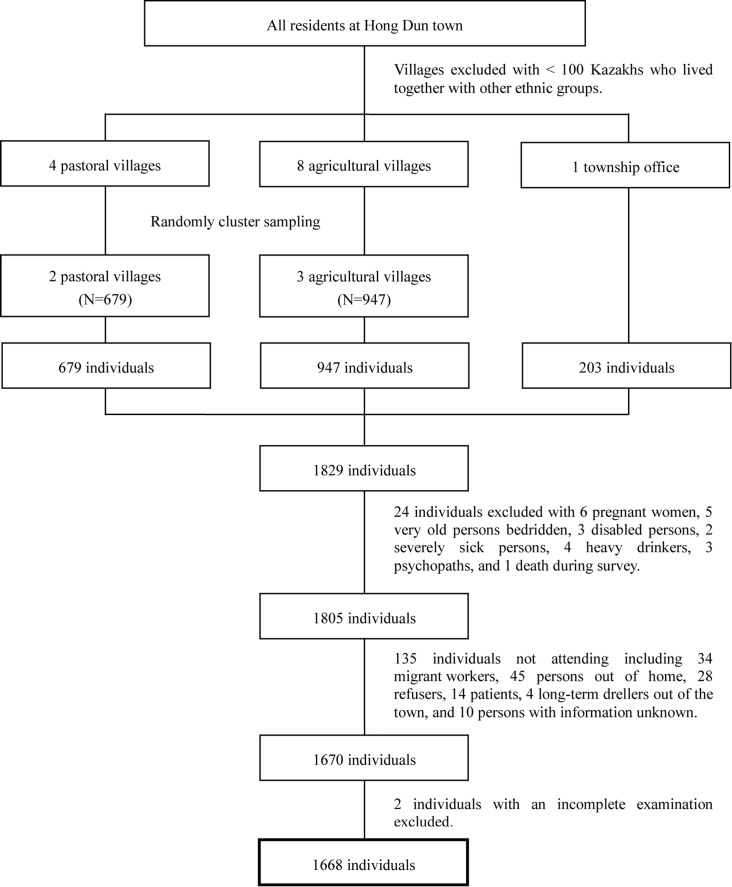
Flow chart for recruitment of participants.

Stratified random-cluster sampling was performed based on a computer-generated allocation algorithm (Fig 1). In sampled units, all Kazakh people aged 30 years or over were recruited for the study with a consideration of sample size based on the prevalence (46.8%) of hypertension among Kazakhs aged 30–70 years [13]. Kazakh was defined as a person whose biological parents were both Kazakh and who had Kazakh paternal/maternal biological grandparents. Pregnant women, persons bedridden aged over 80 years, disabled persons, or persons with mental disorders or other severe disease (as determined by doctors) were excluded. There were 1805 people who met eligibility criteria and 1668 people completed the survey. This represented 92.4% compliance; specifically, 94.4% (637/675) from pastoral villages, 90.2% (838/929) from agricultural villages, and 96.0% (193/201) from the township office.

Between October 2012 and February 2013, participants were invited to take a face-to-face questionnaire survey that included demographic information, hypertensive or CV risk factors, and medical history. Physical examinations, including weight, height, waist, blood pressure (BP) measurements, electrocardiography (ECG), and laboratory tests, were performed. All participants gave their written informed consent to participate in this study whose ethical approval was from the Ethics Committee of Institute of Basic Medical Sciences Chinese Academy of Medical Sciences.

### Acquirement of information on familial aggregation

Familial relationship information was collected in April 2013 based on current living backgrounds, including and limited to consanguineous and marital relationships of three generations within subjects recruited. When a husband and his wife were both oriented from the same village, the couples would be classificated into the husband’s or the wife’s family at random. These relationships were recorded from the highest through the third generation. Members of the highest generation within one family could be couples or siblings with their spouses. Therefore, 303 families that had at least two members and included 1370 family-based subjects were confirmed.

### Assessment of hypertension and related CV risk

After an overnight fasting, BP was measured with an appropriate arm cuff and a mercury column sphygmomanometer on the left arm after a resting period of at least 10 min in the supine position. BP was defined as the mean of two readings, or three readings if there was a difference of more than 5 mmHg between the initial readings [1].

2013 guidelines on hypertension from the European Society of Hypertension and the European Society of Cardiology (ESH/ESC Guidelines) were used at the stage of analysis [10]. Hypertension was defined as a systolic BP ≥ 140 mmHg, a diastolic BP ≥ 90 mmHg, or both, or the use of antihypertensive medications within the last 2 weeks. BP was categorized as optimal, normal, high normal, and Grade 1–3 hypertension, by the highest BP level, whether systolic or diastolic. Moderate-or-high total cardiovascular risk (mTCR) based on BP category, CV risk factors, asymptomatic organ damages (ODs) and presence of diabetes, and symptomatic cardiovascular diseases (CVDs) or chronic kidney diseases (CKDs) was stratified (Table 1) and meant a progressively increasing absolute likehood (≥ 15%) of developing a major CV event within the next 10 years [9,10].

**Table 1:** Definition of moderate or higher total cardiovascular risk.

Other risk factors, asymptomatic ODs or diseases	BP, mm Hg			
	High normal: systolic BP 130–139 or Diastolic BP 85–89	Grade 1 hypertension: systolic BP 140–159 or Diastolic BP 90–99	Grade 2 hypertension: systolic BP 160–179 or Diastolic BP 100–109	Grade 3 hypertension: systolic BP ≥180 or Diastolic BP ≥110
No other RF		Low risk	Moderate risk	High risk
1–2 RF	Low risk	Moderate risk	Moderate to high risk	High risk
≥3 RF	Low to moderate risk	Moderate to high risk	High risk	High risk
OD, CKD stage 3 or diabetes	Moderate to high risk	High risk	High risk	High to very high risk
Symptomatic CVD, CKD stage ≥4 or diabetes	Very high risk	Very high risk	Very high risk	Very high risk

BP: Blood pressure; CKD: chronic kidney disease; CVD: cardiovascular disease; OD: organ damage; RF: risk factor.

We defined male sex, age (men ≥ 55 years; women ≥ 65 years), smoking (at least one cigarette per day), dyslipidemia (total cholesterol [TC] > 4.9 mmol/L, and/or low-density lipoprotein cholesterol > 3.0 mmol/L, and/or high-density lipoprotein cholesterol < 1.0 mmol/L for men and < 1.2 mmol/L for women, and/or triglyceride > 1.7 mmol/L), impaired fasting glucose (5.6–6.9 mmol/L without hypoglycemic drug treatment), and obesity (body mass index [BMI] ≥ 30 kg/m^2^) or abdominal obesity (waist circumference ≥ 90 cm for men and ≥ 85 cm for women [1]) as CV risk factors; pulse pressure (in the persons aged ≥ 60 years) ≥ 60 mmHg, electrocardiographic left ventricular hypertrophy, CKD with eGFR 30–60 mL∙min^-1^∙1.73m^-2^ as asymptomatic ODs; fasting plasma glucose ≥ 7.0 mmol/L or the use of hypoglycemic medications within the last 2 weeks as diabetes mellitus; and stroke from a questionnaire, myocardial infarction from ECG, and CKD with eGFR < 30 mL∙min^-1^∙1.73m^-2^ as established CV or renal disease. Staging of CKD with eGFR was done on the basis of K/DOQI clinical practice guidelines [14].

### Sodium intake and laboratory measurements including UA

Venous blood samples were collected after an overnight fast of at least 10 h, and plasma was immediately separated. Sodium intake was assessed by urinary sodium excretion from the second urine sample after waking, urine creatinine (Cr) concentration, and the 24-h urinary Cr excreted as estimated from height, body weight, and age [15]. Each 43 mmol of sodium is approximately equivalent to 1 g of sodium or 2.5 g of salt (sodium chloride). Plasma UA concentration was measured with the uricase/POD method (Roche Diagnostics, Indianapolis, IN, USA) and a coefficient of variation of 4.0% was achieved. Hyperuricemia was defined as > 416 mmol/L in men and >357 mmol/L in women [16].

All chemistry measurements, including plasma UA concentrations were measured with a Beckman Coulter AU2700 Clinical Chemistry Analyzer (CA, USA). All electrolytes were measured with a Caretium XI-921CT Electrolyte Analyzer (Shenzhen, China).

### Statistical analyses

Prevalence ratios (PRs) with 95% confidence intervals (95% CIs) were used to express associations of plasma UA with hypertension and mTCR. For calculating PRs, robust Poisson method-based models, excluding missing data less than 0.5% of all subjects, and UA quartiles were used. For evaluating effect modification of sodium intake on the associations of UA with hypertension and mTCR, interactions and the main effects of sodium intake and UA were illustrated in robust Poisson models, then tertile of sodium intake and a common reference group, defined as one with the lowest quartile of UA and the lowest tertile of sodium intake, were used in stratified analysis. Families were combined into such Poisson models as repeat measurements [17]. For evaluating familiar intraclass correlation, intraclass correlation coefficients (ICCs), i.e. the covariance parameter estimate for family divided by the sum of the family and residual covariance estimates, were calculated on two-level logistic models where “family” was a random effect or Level 2 unit [18].

SAS version 9.2 (SAS Institute, Cary, NC, USA) was used for all analyses. All *P*-values were two sided except *P* trend tests based on robust Poisson method, in which one-sided *P* values were used, and a *P*-value < 0.05 was considered statistically significant.

## Results

This population had male proportions of 46.7% and average ages of 46.5±12.3 years. The prevalence of hypertension and hyperuricemia was 45.3% and 6.2% (50.1% and 7.6% for men, 41.1% and 4.9% for women). The average salt intake daily of this population is 17.6 ± 14.2 g. Other sex-specific population characteristics were shown in Table 2.

**Table 2:** Study sample characteristics.

	Men (n = 779)	Women (n = 889)	Total (n = 1668)
Age, years	46.5 ± 12.1	46.6 ± 12.5	46.5 ± 12.3
Occupational background, %			
Nomad	38.4	38.0	38.2
Farmer	52.6	48.1	50.2
Non-manual worker	9.0	13.8	11.6
BP variables			
SBP, mm Hg	137 ± 22	135 ± 26	136 ± 24
DBP, mm Hg	87 ± 13	84 ± 13	85 ± 13
Hypertension, %	50.1	41.1	45.3
mTCR, %	55.3	42.9	48.7
Antihypertensive agent, %	8.1	17.4	13.1
UA variables			
UA level, mmol/L	306 ± 74	236 ± 69	269 ± 80
Hyperuricemia, %	7.8	5.1	6.4
Salt intake daily, g	18.9 ± 17.6	16.5 ± 10.1	17.6 ± 14.2
Smoking, %	55.5	2.6	27.3
Drinking, %	28.8	0.6	13.7
BMI, kg/m^2^	25.7 ± 4.3	26.9 ± 5.1	26.3 ± 4.8
TC, mmol/L	5.28 ± 0.95	4.95 ± 0.93	5.10 ± 0.96
Fasting blood glucose, mmol/L	5.33 ± 0.80	5.14 ± 0.74	5.23 ± 0.77
Blood Cr, mmol/L	74.0 ± 11.8	62.3 ± 34.9	67.7 ± 27.4

Values are means ± SD and percent.

Overall family-based ICC values for hypertension and mTCR were 0.041 and 0.033 with a dramatic difference on sex (Table 3). For example, 0.055 of ICC was observed in men; however, this value was approximately equal to zero in women.

**Table 3:** Intraclass correlation of the family based on two-level logistic models.

Dependent variable	Men		Women		Total	
	Random variance	ICC	Random variance	ICC	Random variance	ICC
Hypertension	0.192	0.055	< 0.001	< 0.001	0.141	0.041
mTCR	0.152	0.044	< 0.001	< 0.001	0.113	0.033

ICC: intraclass correlation coefficients; Residual variance: *π*^2^ / 3 = 3.289.

We established sex-specific and age-adjusted simple models only including major effects with their interaction item or not as shown in Table 4. Both UA and salt levels as major effects were correlated with hypertension and mTCR with a statistical significance; their interaction item when being included in the models also showed a statistical or marginal significance. The findings were accordant in multivariable models adjusted for influencing factors such as age, occupation, smoking (only for men), alcohol intake (only for men), BMI, plasma Cr, TC, fasting glucose, and antihypertensive treatment for hypertension and age, occupation, and antihypertensive treatment for mTCR.

**Table 4:** Major effects of UA and salt intake and their interaction.

	Men					Women				
	UA, mmol/L		Salt intake, gram		Interaction	UA, mmol/L		Salt intake, gram		Interaction
	PR	*P* value	PR	*P* value	*P* value	PR	*P* value	PR	*P* value	*P* value
Age-adjusted simple model										
Major effects without interaction item										
Hypertension as dependent variable	1.031	0.004	1.093	0.002	–	1.031	0.004	1.115	< 0.001	–
mTCR as dependent variable	1.024	0.012	1.091	0.001	–	1.028	0.008	1.111	< 0.001	–
Major effects with interaction item										
Hypertension as dependent variable	1.045	0.001	1.004	< 0.001	0.024	1.055	0.005	1.013	0.120	0.132
mTCR as dependent variable	1.039	0.002	1.004	< 0.001	0.019	1.075	< 0.001	1.022	0.002	0.010
Multivariable model										
Major effects without interaction item										
Hypertension as dependent variable*	1.007	0.333	1.067	0.075	–	0.999	0.912	1.107	< 0.001	–
mTCR as dependent variable**	1.024	0.015	1.096	0.014	–	1.021	0.032	1.114	< 0.001	–
Major effects with interaction item										
Hypertension as dependent variable*	1.016	0.242	1.002	0.025	0.129	1.021	0.064	1.013	0.103	0.146
mTCR as dependent variable**	1.034	0.009	1.003	0.004	0.057	1.068	< 0.001	1.023	< 0.001	0.010

UA and salt intake as major effects were included in age-adjusted simple model with interaction item or not. UA level was categorized from its P_25_ with a step of 20 mmol/L. Salt intake daily was categorized from 5 g with a step of 5 g in major effect-based model, however, defined as a continuous variable with a unit of gram in interaction item-based model.

*Adjusted for age, occupation, smoking (only for men), alcohol intake (only for men), BMI, plasma Cr, TC, fasting glucose, and antihypertensive treatment.

**Adjusted for age, occupation, and antihypertensive treatment.–Not applicable. mTCR: moderate-or-high total cardiovascular risk.

Salt-based stratified results with multivariable models, including major effects and other influencing factors, were shown in Table 5. Although no overall association was observed in men, in the highest tertile of sodium intake, the PRs (95% CIs) from low to high quartiles of UA, comparing with lowest quartile of UA, were 1.05 (0.77–1.42), 1.14 (0.82–1.58), and 1.24 (0.94–1.64) for hypertension (*P* for trend = 0.140) and 1.05 (0.82–1.36), 0.98 (0.73–1.32), and 1.28 (0.99–1.65) for mTCR (*P* for trend = 0.035). In women with an overall association, such PRs were 1.22 (0.78–1.91), 1.18 (0.75–1.85), and 1.65 (1.09–2.51) for hypertension (*P* for trend = 0.008) and 1.19 (0.74–1.90), 1.39 (0.91–2.11), and 1.65 (1.10–2.47) for mTCR (*P* for trend = 0.019). However, these significant or marginally significant linear trends, regardless of sex, were not shown in other sodium intake levels.

**Table 5:** Association of UA, modified by sodium intake, with hypertension and mTCR.

	Quartiles of UA with median (interquartile range), mmol/L										
Models	Men					Women					
	227 (203–246)	288 (276–298)	326 (315–341)	391 (375–425)	*P* for trend†	163 (149–175)	212 (200–222)	249 (238–264)	321 (293–353)	*P* for trend†
Adjusted for age only										
Hypertension										
1st tertile of sodium intake	1.00	0.66 (0.43, 1.02)	0.88 (0.61, 1.27)	1.11 (0.81, 1.53)	0.128	1.00	2.26 (1.15, 4.42)	2.39 (1.26, 4.53)	2.27 (1.18, 4.37)	0.033
2nd tertile of sodium intake	1.00	0.95 (0.64, 1.43)	1.01 (0.72, 1.42)	1.10 (0.77, 1.59)	0.125	1.00	1.29 (0.85, 1.96)	1.33 (0.91, 1.94)	1.40 (0.93, 2.11)	0.493
3rd tertile of sodium intake	1.00	1.02 (0.76, 1.37)	1.09 (0.81, 1.47)	1.42 (1.08, 1.86)	0.019	1.00	1.03 (0.65, 1.65)	1.24 (0.80, 1.93)	1.66 (1.10, 1.51)	0.002
Total‡	1.00	0.88 (0.72, 1.09)	0.98 (0.81, 1.20)	1.18 (0.98, 1.40)	0.019	1.00	1.34 (1.01, 1.77)	1.46 (1.12, 1.88)	1.67 (1.31, 2.14)	0.008
mTCR										
1st tertile of sodium intake	1.00	0.63 (0.43, 0.92)	0.83 (0.60, 1.15)	0.98 (0.75, 1.29)	0.414	1.00	2.21 (1.19, 4.12)	2.48 (1.37, 4.48)	2.09 (1.10, 3.95)	0.080
2nd tertile of sodium intake	1.00	0.89 (0.62, 1.29)	1.15 (0.87, 1.51)	1.16 (0.86, 1.57)	0.021	1.00	1.47 (0.97, 2.22)	1.40 (0.97, 2.01)	1.43 (0.96, 2.13)	0.157
3rd tertile of sodium intake	1.00	1.04 (0.81, 1.34)	0.94 (0.70, 1.26)	1.25 (0.96, 1.63)	0.081	1.00	1.08 (0.67, 1.76)	1.36 (0.88, 2.08)	1.62 (1.07, 1.45)	0.009
Total‡	1.00	0.86 (0.72, 1.04)	0.97 (0.81, 1.15)	1.10 (0.95, 1.29)	0.049	1.00	1.44 (1.09, 1.89)	1.57 (1.22, 2.01)	1.64 (1.28, 2.11)	0.001
Adjusted for multivariables										
Hypertension*										
1st tertile of sodium intake	1.00	0.64 (0.42, 0.98)	0.74 (0.51, 1.06)	0.86 (0.64, 1.16)	0.264	1.00	1.85 (1.05, 3.26)	1.62 (0.83, 3.14)	1.65 (0.75, 3.60)	0.120
2nd tertile of sodium intake	1.00	0.89 (0.59, 1.34)	0.94 (0.65, 1.38)	0.84 (0.53, 1.33)	0.200	1.00	1.42 (0.91, 2.21)	1.46 (1.01, 2.11)	1.60 (0.98, 2.63)	0.330
3rd tertile of sodium intake	1.00	1.05 (0.77, 1.42)	1.14 (0.82, 1.58)	1.24 (0.94, 1.64)	0.140	1.00	1.22 (0.78, 1.91)	1.18 (0.75, 1.85)	1.65 (1.09, 2.51)	0.008
Total‡	1.00	0.88 (0.71, 1.09)	0.92 (0.75, 1.13)	0.93 (0.77, 1.13)	0.500	1.00	1.46 (1.11, 1.92)	1.44 (1.11, 1.85)	1.47 (1.13, 1.92)	0.307
mTCR**										
1st tertile of sodium intake	1.00	0.65 (0.44, 0.96)	0.77 (0.57, 1.05)	0.90 (0.70, 1.16)	0.262	1.00	2.12 (1.14, 2.94)	2.30 (1.25, 4.24)	1.98 (0.94, 4.18)	0.330
2nd tertile of sodium intake	1.00	0.90 (0.63, 1.29)	1.25 (0.95, 1.65)	1.19 (0.89, 1.60)	0.006	1.00	1.58 (1.08, 2.32)	1.53 (1.06, 2.19)	1.53 (1.06, 2.22)	0.738
3rd tertile of sodium intake	1.00	1.05 (0.82, 1.36)	0.98 (0.73, 1.32)	1.28 (0.99, 1.65)	0.035	1.00	1.19 (0.74, 1.90)	1.39 (0.91, 2.11)	1.65 (1.10, 2.47)	0.019
Total‡	1.00	0.89 (0.74, 1.07)	0.98 (0.83, 1.17)	1.08 (0.92, 1.26)	0.048	1.00	1.52 (1.18, 1.98)	1.62 (1.26, 2.08)	1.63 (1.24, 2.14)	0.015

*Adjusted for age, residence, smoking, alcohol intake, BMI, urine sodium, plasma Cr, TC, fasting glucose, and antihypertensive treatment.

**Adjusted for age, residence, and antihypertensive treatment. Tertile 1–3 of sodium intake were defined as < 237, 237–346, and >346 mmol/d in men and < 207, 207–309, and > 309 mmol/d in women.

†UA was defined as a continuous variable (mmol/L).

‡Sodium intake (mmol/d) was adjusted.

**Fig 2:**
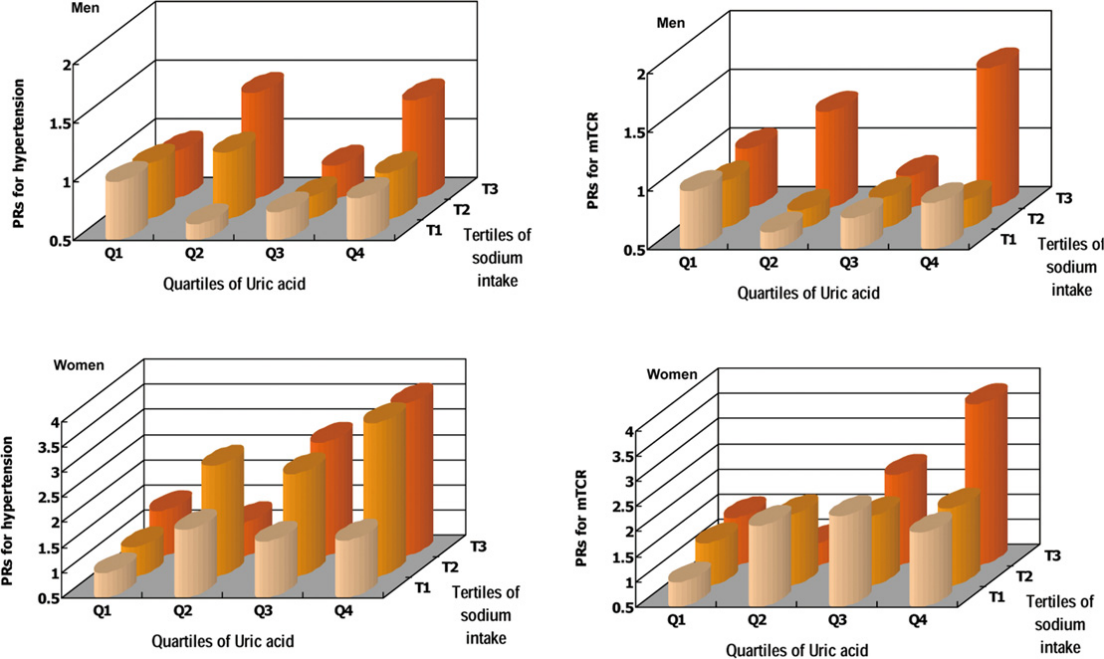
Adjusted PRs for hypertension and mTCR according to multivariables and familial aggregation. A common reference group was used. Multivariables for hypertension were included age, residence, smoking, alcohol intake, BMI, sodium intake, Cr, TC, fasting glucose, and antihypertensive treatment. Multivariables for mTCR were included age, residence, sodium intake, and antihypertensive treatment. Tertile 1–3 of sodium intake were defined as < 237, 237–346, and >346 mmol/d in men and < 207, 207–309, and > 309 mmol/d in women. Quartile 1–4 of plasma UA were defined as <258, 258–301, 302–349, and >349 mmol/L for men; and <188, 188–226, 227–272, and >272 mmol/L for women.

The PRs for mTCR markedly increased with a concomitant increase in UA and sodium intake when comparing with the common reference group, thus the strongest associations were observed with PRs of 1.69 (1.10–2.60) for men and 3.70 (2.09–6.52) for women in the highest quartile of UA and the highest tertile of sodium intake group (Fig 2). Similar findings were observed for hypertension, although the strongest associations were observed in the first or second highest tertile of sodium intake group, with a PR of 1.40 (1.07–1.84) for men and 3.56 (1.37–9.27) for women.

## Discussion

To our knowledge, this study indicated for the first time that a high-salt intake enhanced the associations of blood UA with hypertension and in particular related CV risk. Therefore, if a high-salt diet is not reduced, lowering UA levels may be not enough to prevent hypertension and CV outcomes.

Our analysis showed all of a high UA level, a high-salt intake, and their synergistic action could positively contribute to hypertension and mTCR (Table 4). Up to date, two studies have explored the relationship between UA and sodium metabolism. The first study cross-sectionally indicated that UA levels were inversely and independently associated with proximal tubular sodium excretion, and a greater degree of sodium reabsorbed at nephron sites proximal to the distal tubule was associated with higher circulating levels of UA [19]. The other study, which was conducted in a prospective population, found that a higher sodium intake was associated with greater longitudinal increases in UA [12]. Studies suggested that hyperuricemia is associated with endothelial dysfunction, activation of the renin-angiotensin system (RAS), and pre-glomerular vascular diseases [20]; these likely involve pathogenic mechanisms of salt-sensitive hypertension [11,21]. Also, abnormal renal hemodynamic responses to high-salt intake and alterations of renal sodium handling have been suggested as potential causal mechanisms for the variation in BP responses to sodium [22]. Eventually, salt-sensitive hypertension results in a substantially increased risk of CV events, such as myocardial infarction and stroke of more than twice compared with non-salt-sensitive hypertension [23].

High dietary salt intake may play an important role in the activation of the intrarenal RAS thus facilitating an effect of UA on the intrarenal RAS. This is different from activated circulating RAS mainly during relatively low sodium balance. Increased angiotensin II (Ang II), especially following activation of angiotensinogen locally produced by the proximal tubule cells which was likely not affected by sex and age [22,24], has been suggested to result in the subsequent development of salt-sensitive hypertension in the rat [25]. An updated study by Perlstein *et al* demonstrated that UA level was associated with low basal renal plasma flow and blunted renal vasoconstriction that typically is seen with Ang II infusion during high-salt intake (i.e. ≥ 11.7 g dietary salt per day), lending support to the hypothesis that UA on the condition of a high-salt balance may directly activate the renal RAS thus increase BP [26], but another study conducted in healthy volunteers, who were given 10 g of sodium salt per day on the basis of established salt-sensitive human model, showed a non-significant correlation (*r* = 0.31) between hyperuricemia and salt-sensitivity [27]. Our population-based study likely provides a new line of evidence of high-salt intake that involves into biomedical mechanism of UA associated with hypertension and related CV risk. When 24-h salt intake was up to a higher level (e.g. ≥ 20.2 g for men or ≥ 18.1 g for women), the prevalence of hypertension and long-term likelihood of CV diseases increased with an increased UA level (Table 5); this trend was highly consistent in either men or women, though they showed a dramatically different overall association between UA and hypertension.

Our findings provide some valuable information for clinical practice.

First of all, salt restriction may be considered in preference to lowering UA levels in both male and female populations with a high-salt diet, even if the association of UA with hypertension and mTCR is presently not confirmed. This is especially important for controlling hypertension and CV diseases for China where most people have an average daily salt-intake of over 12 g [11]. In particular, people of ethnic minorities, such as Kazakhs, Tibetan, and Mongolian, with a high prevalence of hypertension have a fairly high-salt intake due to traditional lifestyle. For instance, 1991 National Hypertension Survey displayed that Kazakhs had the highest female prevalence and the forth highest male prevalence of hypertension. Previous studies showed that diet salt intake of Kazakhs varied from 12.4 g to 40 g per day due to different survey methods and population variation [28,29]. Traditionally, their main diet, with limited fruit and vegetable daily, is milk tea, dried meat, and cooked wheaten food, all of which are added much salt. This is similar to the diet in industrialized countries that possesses a characteristic of high-sodium and low-potassium, which is closely associated with hypertension [30]. Furthermore, studies showed diet salt intake daily of Altay Kazakhs seemly increased 3.5 g within 12 years from 24-h urine-based 12.4 g of the WHO Cardiovascular Diseases and Alimentary Comparison Study (also conducted in Altay) to 15.9 g of ours [30], which was much higher than salt intake level in industrialized countries such as 9–12 g per day in European countries [10]. As a result, salt-restriction could be the most pressing problem to control hypertension among Kazakhs.

For another, the treatment of asymptomatic hyperuricemia is presently under debate. Obviously, this study does not contribute any information to the treatment of asymptomatic hyperuricemia in men; even for women, salt-restriction should be considered prior to the treatment of asymptomatic hyperuricemia.

Multiple strengths lie in this study. First, this study was based on a high-quality design and performing, included a selection of the Kazakh people with a daily high-salt intake as our study population, a 92.4% compliance of a random sample, and a population setting covering pastoral, agricultural, and urban areas that meant good generalization. Second, we adjusted for familial aggregation, besides multiple confounding factors, to improve precision for estimating effects. Finally, the long-term risk of UA on CV outcomes related to hypertension was evaluated by using risk stratifications from ESH/ESC Guidelines, although the Seventh Report of the Joint National Committee on Prevention, Detection, Evaluation, and Treatment of High Blood Pressure (JNC 7), different from the JNC 6, abandoned this stratification, it is still believed to be helpful for clinical practice and etiological studies by the WHO and Chinese Hypertension League (CHL) [1,9].

This study has some potential limitations. In the context of cross-sectional design, temporality is difficult to establish in this study, so prospective studies are required to examine our results. 24 h urine collection was not used, but the second urine sample after waking here was thought as a satisfactory alternative to 24-h pooled urine in adults for extensive epidemiological surveys [15,31] and used in a large scale epidemiological field [32].

Overall, this study presented and probed into a hypothesis of enhancement from a high salt intake on associations of UA with hypertension and a progressively increasing long-term absolute likelihood of developing a major CV event. In any case, salt-restriction, in preference to the treatment of asymptomatic hyperuricemia, may be recommended among high-salt diet populations such as Kazakhs, though this requires a specific randomized controlled trial.
